# A Proteomic Approach Provides New Insights into the Control of Soil-Borne Plant Pathogens by *Bacillus* Species

**DOI:** 10.1371/journal.pone.0053182

**Published:** 2013-01-03

**Authors:** Ömür Baysal, Duo Lai, Han-Hong Xu, Mirko Siragusa, Mikail Çalışkan, Francesco Carimi, Jaime A. Teixeira. da Silva, Mahmut Tör

**Affiliations:** 1 Department of Molecular Biology and Genetic, Faculty of Life Sciences, Muğla Sıtkı Koçman University, Muğla, Turkey; 2 Key Laboratory of Natural Pesticide and Chemical Biology of Ministry of Education, State Key Laboratory for Conservation and Utilization of Subtropical Agro-bioresources, South China Agricultural University, Guangzhou, P. R. China; 3 CNR, Research Division Palermo, Institute of Plant Genetics, Palermo, Italy; 4 Central Research Institute for Field Crops, Turkish Ministry of Food, Agriculture and Livestock, Ankara, Turkey; 5 Faculty of Agriculture and Graduate School of Agriculture, Kagawa University, Miki cho, Kita gun, Ikenobe, Japan; 6 National Pollen and Aerobiology Research Unit (NPARU), The University of Worcester, Henwick Grove, Worcester, United Kingdom; University of Illinois at Urbana-Champaign, United States of America

## Abstract

Beneficial microorganisms (also known as biopesticides) are considered to be one of the most promising methods for more rational and safe crop management practices. We used *Bacillus* strains EU07, QST713 and FZB24, and investigated their inhibitory effect on *Fusarium*. Bacterial cell cultures, cell-free supernatants and volatiles displayed varying degrees of suppressive effect. Proteomic analysis of secreted proteins from EU07 and FZB24 revealed the presence of lytic enzymes, cellulases, proteases, 1,4-β-glucanase and hydrolases, all of which contribute to degradation of the pathogen cell wall. Further proteomic investigations showed that proteins involved in metabolism, protein folding, protein degradation, translation, recognition and signal transduction cascade play an important role in the control of *Fusarium oxysporum*. Our findings provide new knowledge on the mechanism of action of *Bacillus* species and insight into biocontrol mechanisms.

## Introduction

The most prevalent soil-borne pathogen *Fusarium oxysporum* causes serious losses in protected agricultural production areas all over the world. Since control of the pathogen is not possible or is very difficult using chemicals and cultural methods, it is imperative to find an effective control method. Infectious diseases have long been a major threat to food security directly relevant to a world population that has been growing at an annual rate of 1.2%, i.e. 77 million people per year [1]. Accessing adequate amounts of nutritious, safe, and culturally appropriate foods that are produced in an environmentally sustainable manner is important [1]. Pesticide use also plays a major role in modern agriculture, and has contributed to dramatic increases in crop yields over the past four decades for most field-grown fruit and vegetable crops. Nevertheless, these chemicals also pose some major risks if used improperly or too frequently. Many pesticides, even when applied legally and according to the label’s instructions, may leave residues in or on treated fruits, vegetables, and grains as well as in soil [2]. The development of environmentally friendly alternatives to counter the extensive use of chemical pesticides is one of the biggest ecological challenges facing microbiologists and plant pathologists for combatting crop diseases. Beneficial microorganisms (also known as biopesticides) are considered to be one of the most promising methods for more rational and safe crop-management practices [3]. There are also a number of plant diseases for which a chemical solution is ineffective or non-existent. This, together with an increasing demand by consumers and the public for pesticide-free food, has driven the demand for biological control through the use of natural antagonistic microorganisms.

There is a large body of literature reporting the potential use of rhizosphere-associated bacteria in stimulating plant growth and biocontrol agents [4], [5], [6] for multiple plant diseases caused by soil-borne [7], [8], [9] or post-harvest pathogens [8], [9]. One of the most commonly used and well-studied organisms, the rhizobacterium *B. subtilis*, has an average of 4–5% of its genome devoted to antibiotic synthesis and has the potential to produce more than two dozen structurally diverse antimicrobial compounds [10]. *Bacillus*-based products represent about half of the commercially available bacterial biocontrol agents [11].

*B. subtilis* strains produce a broad spectrum of bioactive peptides with great potential for biotechnological and biopharmaceutical applications. A well-known class of such compounds includes the lipopeptides surfactin [5], fengycin [6], and the iturin compounds including iturin A [6], B, and C [7], mycosubtilins [12], and bacillomycins [12], which are amphiphilic membrane-active biosurfactants and peptide antibiotics with potent antimicrobial activities. *B. subtilis* has also shown particular utility in oil recovery [13], remediation of soil contaminated by heavy metals [14] and biocontrol against phytopathogens [15] and insects [16].

Besides antagonistic peptide antibiotics, other compounds derived from *Bacillus* species play a role as ‘immuno-stimulators’ by reinforcing host resistance. Lipopeptides from *B. subtilis* are synthesized by non-ribosomal peptide synthetases or hybrid polyketide synthases and non-ribosomal peptide synthetases. These modular proteins are responsible for the biosynthesis of several hundred bioactive compounds [17]. However, it remains unclear whether the protective effect is only due to the direct bactericidal activity of *Bacillus* species or if it also indirectly depends on other new molecules showing similarity to known proteins. There is also no strict correlation between biocontrol function of *B. subtilis* isolates and their genotypic and phenotypic variations [18]. Moreover, 30% of the predicted genes present in type strain 168 are also absent in the genomes of other isolates. A relatively recent study indicated that the function of a large number of genes in *B. subtilis* remains unknown [19].

In our previous study, we tested two *B. subtilis* strains, including QST713 and EU07, on *Fusarium oxysporum* f.sp. *radicis-lycopersici* [*FORL*] [20]. We showed the existence of a higher inhibition effect of EU07 on *FORL* compared to QST713, which was correlated with a significant increase in plant height of EU07-treated plants [20].

In the present study, we aimed to elucidate the reasons for one biocontol agent’s superiority to the other in controlling *Fusarium oxysporum* f. sp. *radicis-lycopersici* (FORL).

## Results

### *B. subtilis* Strains have a Suppressive Effect on FORL

**Figure 1:**
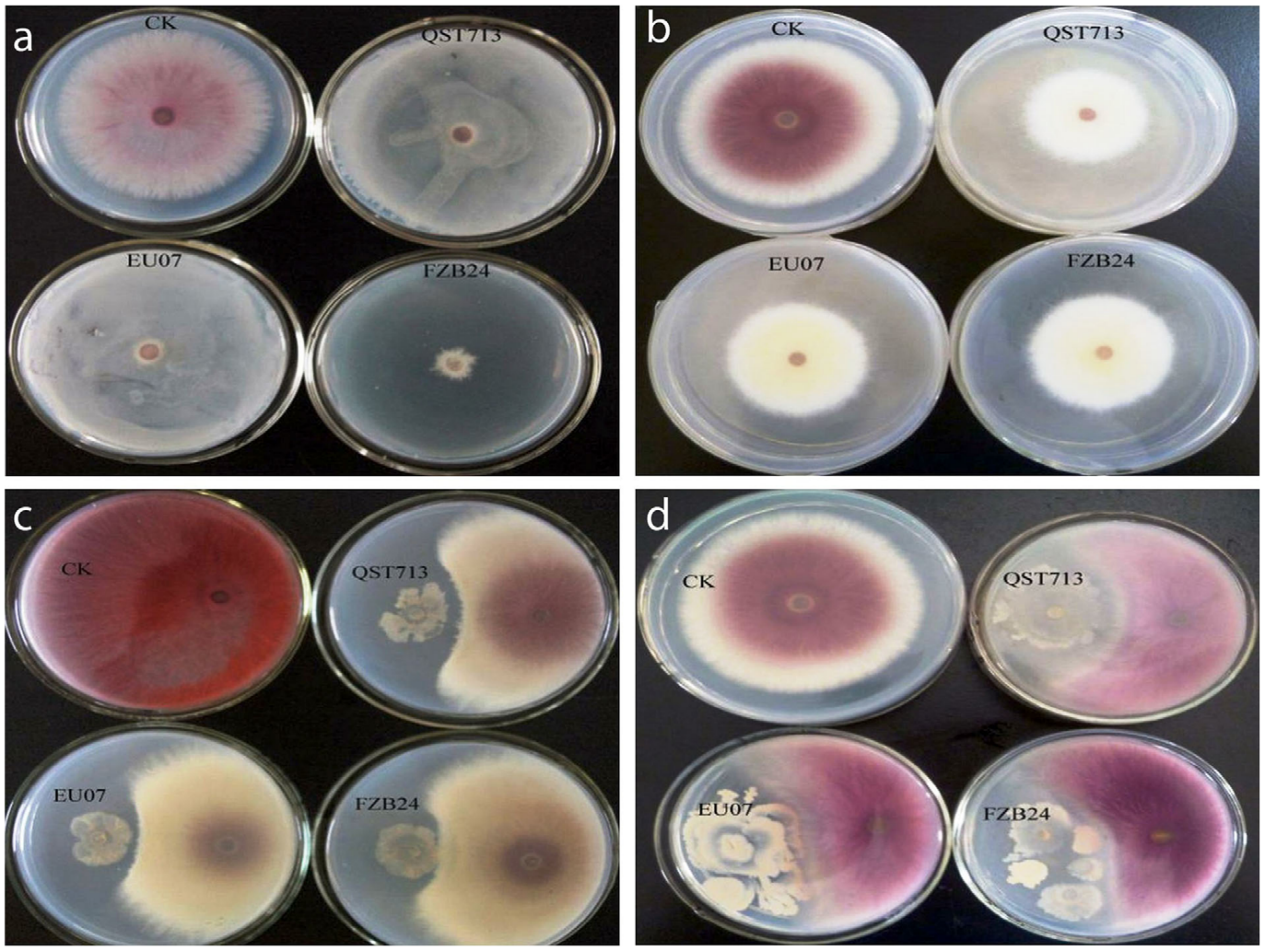
Antagonistic effect of three *Bacillus* strains *in vitro*. a) Three-layer agar assays: Upper to lower is FORL, *Bacillus* and PDA. b) Sealed dish assays: Upper dish inoculated FORL, lower dish inoculated *Bacillus* strains. c, d) Testing inhibitory effect by reciprocal inoculations.

The antagonistic effect of three *B. subtilis* strains, FZB 24 and QST713 and EU07, on FORL was investigated. All strains showed equal inhibitory effects in culture medium (Fig. 1). Interestingly, the inhibitory effect of *Bacillus* strains was visible on Petri dishes in three-layer agar assays (Fig. 1a), sealed assays (Fig. 1b) and reciprocal inoculations (Fig. 1 c, d). Although the inoculations were performed at the same time, strain EU07 grew much faster than the other two commercial strains (Fig. 1d).

### *B. subtilis* Strains Show Genetic Variation

**Figure 2:**
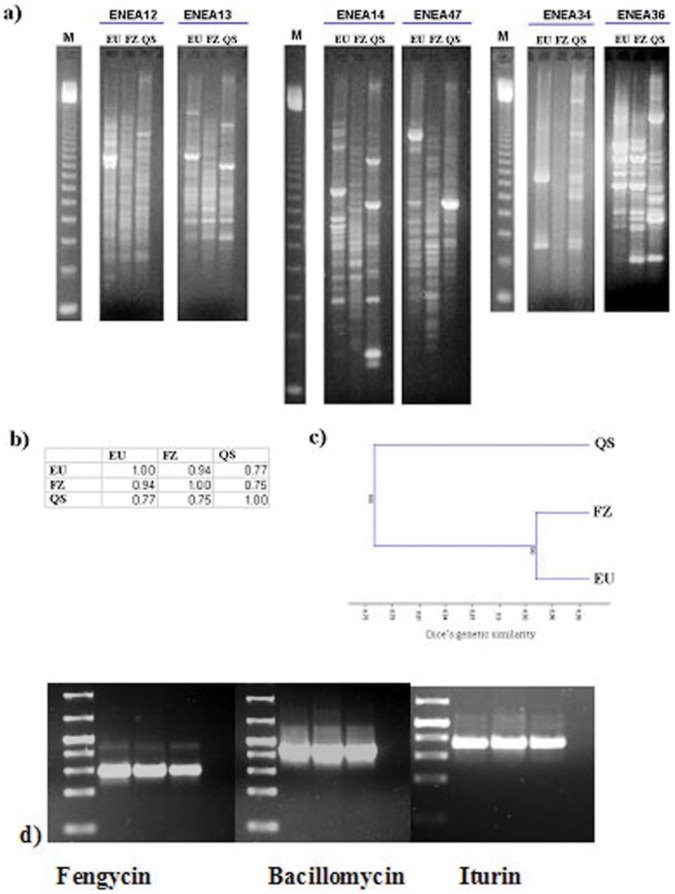
Molecular analysis of QST713, EU07 and FZB24, and their genetic comparison with antibotic genes in three strains. Fengycin (Fen D), bacillomycin Bmy A and Iturin (Itu C) genes were amplified from the three strains EU07, QST713, FZB24. The same results on PCR amplifications were also observed with three other independent replicates.

Since there was no remarkable difference in their inhibition on FORL, we investigated their genetic relationship. Their genetic discrimination was carried out using the 6 inter-simple sequence repeat (ISSR) primers which showed the highest polymorphisms within tested 26 different ISSR markers tested (Fig. 2a). A total of 62 well-resolved band classes were detected and used for genetic analysis. The analysed amplified fragments ranged from 150 bp [primer ENEA 14] to 2.3 Kbp [primers ENEA13 and 14] in size. The number of ISSR bands obtained for each primer varied from 8 [primer ENEA12] to 15 [primers ENEA13 and 14], with an average of 10.3 bands per primer. All the primers screened revealed marked polymorphisms in all strains, showing a clear genetic differentiation. Out of 62 bands considered, 26 were polymorphic (Pp = 41.9%). The polymorphisms identified were used to generate a Dice’s genetic similarity matrix (Fig. 1b). FZB24 and EU07 showed the highest genetic similarity value (D_EU-FZB24_ = 0.94), while strain QST713 appeared genetically more distant (D_QST713-EU07_ = 0.77; D_QST713-FZB24_ = 0.75). Dice’s similarity was also used to carry out a cluster analysis and to generate a dendrogram showing the relationship among the selected strains, as shown in Fig. 2c. Three different antibiotic genes including (Table S1) fengycin (Fen D), bacillomycin (Bmy A) and iturin (Itu C) genes were also screened by specific PCR amplifications in three different *Bacillus* strains (Fig. 2d).

### Enzymes and Temperature Affect the Inhibitory Activity of EU07 against FORL

**Figure 3:**
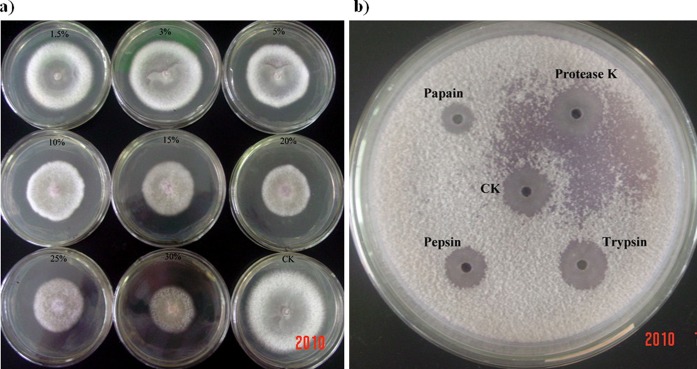
Effect of enzymes and temperature on the inhibitory activity of cell-free supernatant of strain EU07 against FORL. a) The cell-free supernatant from strains EU07 showed visible inhibition effects on the mycelial growth. b) Measuring the diameter of inhibition zone (DIZ), the inhibition activity was significantly reduced by papain (DIZ = 14.0 mm) and pepsin (DIZ = 19.0 mm) treatment, but not by proteinase K (DIZ = 24.0 mm) and trypsin (DIZ = 23.5 mm) treatment, comparing to the control CK (DIZ = 24.0 mm).

QST713 and FZB24 are commercially available strains and their inhibitory effects to plant pathogens have already been studied. These strains are registered as the biocontrol products Serenade® (Agraquest) and Taegro® (Novoenzymes Biologicals Inc.). We determined how EU07 compared against the commercial strains in terms of its inhibitory activity on the fungal pathogen, FORL. The cell-free supernatants of EU07 had an effective antifungal function and contained some biologically active constituents against FORL (Fig. 3a). This inhibitory effect against FORL was linked to the concentration of the cell-free supernatant used, and even the 1.5% culture supernatant inhibiting the mycelial growth of FORL. Antifungal activity of the cell-free supernatant remained stable after heating at 60, 90 and 100°C for 1 h (The diameters D of fungal mycelium were 63.8 mm, 64.5 mm and 64.8 mm, respectively. D_CK_ = 74.0 mm ). However, this activity was lost after incubation of the supernatant at 121°C for 30 min (D_121_ = 73.0 mm), indicating that the biologically active components in the cell-free supernatant partly possessed temperature-sensitive properties. When the diameter of inhibition zone (DIZ) was measured, we observed that the inhibitory activity was significantly reduced by papain (DIZ = 14.0 mm) and pepsin (DIZ = 19.0 mm) treatment, but not by proteinase K (DIZ = 24.0 mm) and trypsin (DIZ = 23.5 mm) treatment, relative to the control CK (DIZ = 24.0 mm) (Fig. 3b). These results suggest that the inhibitory activity of the cell-free supernatant is partly dependent on proteinase activity.

### Volatiles from Three *B. subtilis* Strains have Antifungal Activity against FORL

**Figure 4:**
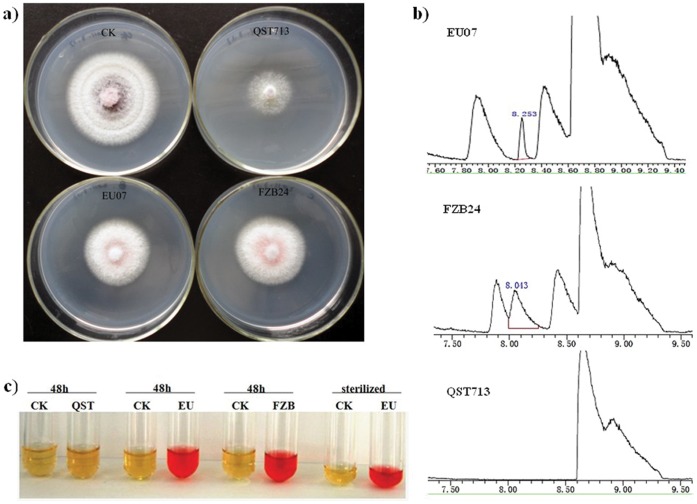
Antifungal activities of volatiles from *B. subtilis* against FORL in sealed plates. a) Volatiles produced by strains EU07, FZB24 and QST713 showed visible inhibition effects on the mycelial growth. b) SPME gas chromatograms of volatiles from strains EU07, FZB24 and QST713, respectively. Volatile production 2,3-butanediol was detected from strains EU07 and FZB24, but not in strain QST713. c) Voges-Proskauer assay of strains EU07, FZB24 exhibited pink color indicating that they produce acetoin, which is not detected in strain QST713.

**Figure 5:**
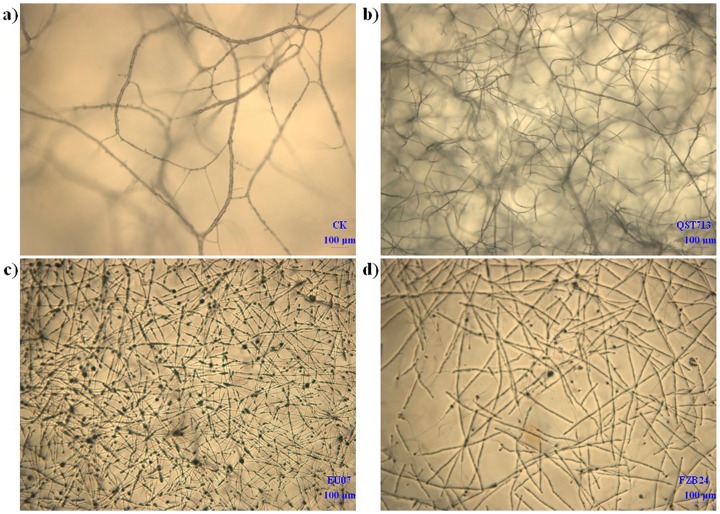
Effects of volatiles from *B. subtilis* on mycelial growth observed under optic microscope. a) Normal mycelia of control treatment. b) Wizened mycelium after treatment with strain QST713. c) and d) Morphological aberrance such as irregular distorted, disrupted, wizened and swelled mycelium after treatment with strains EU07 and FZB24.

All three *B. subtilis* strains (EU07, FZB24, QST713) produced antifungal volatile compounds (VOCs) and inhibited the mycelial growth of FORL by 26.2, 23 and 44.3%, respectively (Fig. 4a). VOCs from all three *B. subtilis* strains were determined by SPME-GC/MS. EU07 produced nearly equal 2,3-butanediol with FZB24, while this metabolite was not detected in QST713 (Fig. 4b). 2,3-Butanediol was further confirmed by the Voges-Proskauer assay in which EU07 and FZB24 exhibited a pink color indicating that they produced its precursor metabolite 3-hydroxy-2-butanone (acetoin), which was also not detected in QST713 (Fig. 4c). It is likely that acetoin and 2,3-butanediol were in part the bacterial components responsible for air-borne chemical signaling in EU07 and FZB24 to trigger growth promotion and induced systemic resistance (ISR) response in tomato [21]. After the antifungal-volatile bioassay, the mycelial morphology was observed under an optical microscope. The FORL mycelium treated with EU07 and FZB24 VOC exhibited morphological aberrations such as irregular, distorted, disrupted, shrivelled and swollen mycelia (Fig. 5c, d). No such changes were noticed in control mycelia (Fig. 5a), and only wizened FORL mycelium was observed in the QST713 treatment (Fig. 5b). EU07 (Fig. 5c) had a greater effect on mycelial morphology at the expanded end of the mycelium than FZB24 and QST713 (Fig. 5b, c, d).

### LC-ESI-MS/MS Analysis of Secretory Proteins from EU07 and FZB24

**Figure 6:**
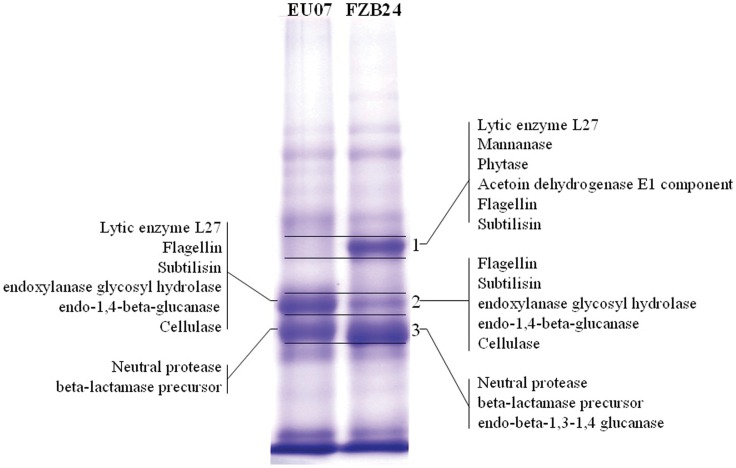
Characterization of secretory proteins using LC-ESI-MS/MS. Left lane: EU07 proteins, right lane: FZB24 proteins, the numbering of the 3 excised gel slices is shown on the right. The representative non-redundant protein identifications are shown on the gel slice.

Secretory proteins in the cell-free supernatant of EU07 and FZB24 were separated by SDS-PAGE and the main protein bands of the two stains were visibly different (Fig. 6). The bands of interest in each lane were cut into three sections for in-gel digestion, and tandem MS/MS data was used in Mascot searches. By searching the *B. subtilis* tryptic peptide database (obtained from NCBI), 37 proteins from EU07 and 43 proteins from FZB24 were identified (*p*<0.05) (Table S2). Of 37 proteins from EU07, 20 had a notably high identification score (>100), most of them being assigned to a protease function, which corresponds with the inhibitory activity of the cell-free supernatant being partly dependent on proteinase activity. Within these secretory proteins, lytic enzyme (gi/3986320 Acc.) received the highest score with 50.91% coverage followed by endoxylanase glycosyl hydrolase familly11 proteins, endo 1,4-β-glucanase, cellulase and neutral protease (Table 1).

**Table 1:** Characterization of secretory proteins of EU07 using LC-ESI-MS/MS.

Accession	Score	Unique peptides	Coverage(%)	MW (kDa)	calc. pI	Description
gi3986320	5252.42	9	50.91	27.5	7.14	lytic enzyme L27 [*Bacillus subtilis*]
gi14278900	15878.87	14	41.83	32.6	4.91	flagellin [*Bacillus subtilis*]
gi14278871	14065.11	14	34.83	35.6	5.25	flagellin [*Bacillus subtilis*]
gi494578	2308.40	6	31.64	27.5	6.80	Subtilisin
gi257062286	123.13	2	15.02	23.3	9.50	endoxylanase glycosyl hydrolase family 11 protein [*Bacillus subtilis*]
gi110612130	709.87	5	14.63	55.2	7.85	endo-1,4-beta-glucanase [*Bacillus subtilis*]
gi154183741	712.87	5	14.63	55.0	7.59	cellulase [*Bacillus subtilis*]
gi226529	34.62	1	6.00	32.7	5.87	neutral protease
gi320017802	85.22	1	3.62	33.2	8.81	beta-lactamase precursor [*Bacillus subtilis BSn5*]

### Identification of Differentially Expressed Proteins of *Bacillus subtilis* EU07 and FZB24

The proteins in EU07 and FZB24 cells were separated by IEF as the first dimension and by SDS-PAGE as the second dimension followed by CBB R-250 staining. The protein profiles of 2-D PAGE were obtained by scanning, digitizing, and image analysis (Figure S1). More than 3,500 protein spots were detected on each of the 2-D gels. Comparative analysis of the 2-D electrophoresis between EU07 and FZB24 revealed 48 spots with 4-fold differential expression. The 42 most significant proteins were identified as either up- or down-regulated in EU07 relative to FZB24 by peptide mass fingerprinting using MALDI-TOF MS analysis and the MASCOT search engine. The expression patterns of proteins shown in Table S2 provides a list of identified proteins with their respective spot ID, up- (↑) or down-(↓) regulated, protein name, NCBI ID, Mascot score, number of matched peptides, functions of identified proteins, percentage of sequence coverage, and the theoretical pI/MW (Table S2). Additionally, the locations of identified proteins on the 2-D gels were labeled with a spot ID (Table 2, Figure S1). The differentially expressed proteins identified in this study were found to be associated with metabolism (carbohydrates and amino acids; 2952, 2964, 3010, 3050, 3164, 3168, 3229, 3238 IDs), protein folding (3000 ID), protein degradation (3180, 3189 IDs), translation (3162 ID), signal transduction (3562 ID), DNA repair, energy production, and protein folding (3475, 2975 ID).

**Table 2:** The homologues of unknown proteins.

Spot no.	Acc. no.^a^	Homologue				
		Acc. no.^b^	Protein name	Species	Ident.^dc^ (%)	Simil.^ed^(%)
2964	gi|154686192	NP_389784.1	Putative phage-related pre-neck appendage protein	*Bacillus subtilis* subsp.	81	100
3318	gi|154686778	YP_896612.1	NIF3-related protein	*Bacillus thuringiensis* str.	63	100
3513	gi|154686192	NP_389784.1	putative phage-related pre-neck appendage protein	*Bacillus subtilis* subsp.	81	100
3766	gi|154685486	NP_388915.1	phosphatase	*Bacillus subtilis* subsp.	71	98
3812	gi|16080155	not finded				
3819	gi|154687215	not finded				
3835	gi|154685032	ZP_01464518.1	S-adenosylmethionine (SAM)-dependent methyltransferase	*Stigmatella aurantiaca*	51	99

BLASTp (http://www.ncbi.nlm.nih.gov/BLAST/) was used to search for homologues of the unknown proteins (Table 1). The homologues with the highest homology are shown.

a Acc. no., The accession number of the unknown proteins in Table 1.

b Acc. no., The accession number of the homologues.

c Ident., identities.

d Simil., similarity.

Here, we report that EU07 has a higher suppressive effect on FORL than FZB24 which is a registered Bacillus strain recommended for the control of soil borne pathogens.

EU07 also expresses new unknown proteins displaying similarity to putative phage-related protein, pre-neck-related protein, phosphatase, S-adenosylmethionine (SAM)-dependent methyltransferase (Table 2), which are of importance in signal transduction and recognition mechanisms in the control of pathogenic soil-borne fungi such as FORL.

## Discussion

We have taken a molecular and proteomic approach to gain insight into the interaction between the biocontrol agent *B. subtilis* and one of its hosts, the soil-borne plant pathogen FORL. Here, we present evidence that: i) *B. subtilis* displays genetic variation; ii) inhibitory products from *B. subtilis* are influenced by enzymes and temperature; iii) VOCs produced by *B. subtilis* have an inhibitory effect on FORL; iv) the majority of the secreted proteins from *B. subtilis* may function as proteases; v) the bioactive compounds are modulated. It has been known that lipopeptides from *B. subtilis* play an important role in biocontrol agent -pathogen interactions [12]. However, our investigation clearly idenitifed new proteins displaying similarity to putative phage-related protein, pre-neck-related protein, phosphatase, and S-adenosylmethionine (SAM)-dependent methyltransferase, which may play a significant role in the signal transduction and recognition mechanism in the control of pathogenic fungi.

Specific agents must compete with other soil- and root-associated microbes to survive, propagate, and express their antagonistic potential during those times when the targeted pathogens pose an active threat to plant health [15], [22]. We used a PCR approach to differentiate QST713, FZB24 and EU07 and showed their genetic relation. We have seen also a genetic correlation between FZB24 and EU07. Since there is no strict correlation between biocontrol function of *B. subtilis* isolates and their genotypic and phenotypic variations [19], it is difficult to suggest that these genetic differences are sufficient to cause the differences in antifungal metabolites. In contrast, general suppression of a plant pathogen is more frequently invoked to explain the reduced incidence or severity of plant diseases because of the activities of multiple organisms that can contribute to a reduction in disease pressure [15], [23].

In general, soil-borne pathogens such as *Fusarium* species that infect through mycelial contact are more susceptible to competition from other soil- and plant-associated microbes than those pathogens that germinate directly on plant surfaces and infect through appressoria and infection pegs [24]. Our results showed that EU07, QST713 and FZB24 treatments of plants suppressed FORL under controlled conditions. However, this suppression was much more expressed with EU07 than that with QST713 and FZB42, which may be attributed to the number and amount of secreted inhibitory molecules.

Our data showed unique secreted peptides including lytic enzyme, endoxylanase glycosyl hydrolase family 11 protein, endo-1,4-β-glucanase, flagellin, subtilisin, cellulase, neutral protease, β-lactamase precursor (Table 2). Enzymes including chitinase, β-1,3-glucanase and cellulase have been reported to be involved in the antagonistic action of *Pseudomonas* against fungal pathogens [25]. These secreted molecules may give an advantage to EU07 over other strains in suppressing the growth of *Fusarium* species.

Antifungal agents produced by microorganisms may be used as effective biocontrol agents instead of microorganisms. Most of the known antifungal agents produced by *B. subtilis* are polypeptides [23], including iturins A–E, and bacillomycins D, F and L [6], [12]. These antifungal peptides inhibit the growth of a large number of fungi, including *Aspergillus*, *Penicillium* and *Fusarium* species [26], [27].

Investigation into VOCs showed the production of 3-hydroxy-2-butanone (acetoin) by EU07 and FZB24 but not by QST713. It has been observed that bacterial VOCs (acetoin or 3-hydroxy-2-butanone) can serve as agents for triggering growth promotion in *Arabidopsis thaliana* [26], [27]. Previously, we reported similar observations in which EU07 treatment of plants resulted in increased plant height in comparison to that observed with QST713 [20]. External applications of commercial acetoin and 2,3-butanadiol, both of which were produced by the two *Bacillus* species, resulted in a dose-dependent stimulation of plant growth under optimum concentrations [28]. In addition, the production of acetoin and 2,3-butanediol by plant growth-promoting bacteria (PGPR) was reported to increase systemic disease resistance and drought tolerance [29], [30].

Our MALDI TOF-MS analyses showed that genes related to the GacA system may be involved in the *Bacillus*-FORL interactions (Table S2). GacA has been previously suggested to be an essential system for the synthesis of extracellular protease and secondary metabolites [31]. The GacS/GacA system was also shown to be associated with the production of antibiotic compounds [32].

Methylation of small molecules and macromolecules is crucial in metabolism, cell signaling, and epigenetic programming and is most often achieved by SAM-dependent methyltransferases [33], [34]. In a recent study, SAM synthetase and other methyl transferases were identified to play a role in phenylpropanoid biosynthesis and in signal transduction on another soil-borne pathogen, *Fusarium oxysporum* f. sp. *cubense* (Foc) [35], [36]. Our investigations into differentially expressed proteins showed that the protein 3835 ID has high similarity (99%) to SAM-dependent methyltransferases (Table 2). Therefore, it is plausible that this protein (3835 ID) may be involved in the interaction between *B. subtilis* and FORL.

It is known that 1-aminocyclopropane-1-carboxylate (ACC) synthase converts S-adenosylmethionine (AdoMet) into ACC, which is thereafter converted to ethylene by ACC oxidase. It has been proposed that PGPR may enhance plant growth by lowering a plant’s ethylene levels. In these cases, the immediate precursor of ethylene is ACC. This compound is hydrolyzed by bacteria-expressing ACC-deaminase activity [35], [36]. Although the details of the molecular interactions are not known, the ethylene and cytokinin signaling pathways appear to be involved in growth promotion induced by VOCs [37]. Since our detected up-regulated protein 3835 ID showed similarity to SAM-dependent methyltransferases, it could be possible that the same mechanism in the inhibition of FORL growth by EU07 may exist. It is postulated that AdoMet is involved in an on-going competition between FORL and EU07. There is also up regulated two-response regulator protein (3562 ID) related to signal transduction, which shows that signalling is active.

Furthermore, our MALDI TOF-MS analysis revealed that one of the upregulated proteins has a high similarity to NIF3-related proteins. In prokaryotes, NIF3 proteins have been reported to be involved in transcriptional regulation [38]. This regulation can be suggested to have a stimulatory effect on generation of the lytic enzyme subtilisin, endoxylanase glycosyl hydrolase familly 11 protein, endo-1,4-β-glucanase, and cellulase-neutral protease (Table 1), all of which have secondary roles as FORL inhibitors. These findings are associated with our present MALDI TOF-MS findings related to 20 with notably high identification scores (>100), most of which have been assigned to a protease function.

Overall, NIF3 proteins, putative phage-related protein, pre-neck-related protein, phosphatase, and SAM-dependent methyltransferase are highly likely to be responsible for pathogen inhibition in the early phase of recognition in addition to the known cell wall-degrading enzymes, acetoin and other components. The present study demonstrates that EU07 is an original *B.* *subtilis* isolate and can suppress FORL growth in tomato plants better than the commercially available registered strains QST713 and FZB24.

Furthermore, we believe that our findings are of importance in view of further experimental applications related to not only agricultural but also medical and pharmacology sciences.

## Materials and Methods

### *In vivo* Bioassays of Pathogen Inhibition Effects of *Bacillus subtilis*

*Bacillus* strains were cultured, activated and the existence of antibiotic genes were also screened as described previously [20]. The pathogen *Fusarium oxysporum* f. sp. *radicis lycopersici* (FORL) was also confirmed with species specific-PCR [22].

*In vivo* antagonism bioassays were carried out to evaluate the pathogen inhibitory effects of *B. subtilis* strains (EU07, FZB24 and QST713). In experiment 1, 100 µL of bacterial culture was incubated over the surface of a potato dextrose agar (PDA, Merck) plate. Simultaneously, a 5-mm mycelial plug cut from the edge of a seven day-old culture of the fungal strain was placed at the center of the plate. In experiment 2, a 5-mm bacterial culture plug cut from *Bacillus* strains were incubated at one side of the Petri dish (3 cm from the center). At the same time, a 5-mm mycelial plug cut from the edge of a seven day-old culture of the fungal strain was placed at the center of the plate. All the plates were incubated at 28°C for 5 days and examined for evidence that growth of the fungus was inhibited by the bacterium. A positive response was the visible zone of inhibition around the fungus.

### Molecular Analysis of QST 713, EU07 and FZB24, and their Genetic Comparison

Genomic DNA was extracted from bacterial culture as described previously [20]. Bacterial colonies were collected into separate tubes. DNA was quantified by measuring as described previously [20]. Of 24 ISSR primers, six were selected showing high polymorphism (ENEA12, ENEA13, ENEA14, ENEA34, ENEA36 and ENEA36) and used to amplify the DNA. The primers were purchased from MWG-Biotech AG, Ebersberg, Germany. Each ISSR amplification was performed in 25 µL containing 20 mM Tris-HCl (pH 8.4), 50 mM KCl, 2 mM MgCl_2_, 800 µM dNTP, 0.5 µM of each primer, 1 U of Platinum *Taq* polymerase (Invitrogen, Life Technologies) and 15 ng of template DNA. PCR was performed in a 96-well GeneAmp® PCR System 9700 (Applied Biosystems) equipped with a hot bonnet under the following cycle program: initial denaturation step for 4 min at 94°C, followed by 36 cycles at 94°C for 30 s, 55°C for 45 s and 72°C for 120 s, followed by a final extension step at 72°C for 7 min. Amplified bands from each primer were scored as present (1) or absent (0). Only those bands that consistently amplified for three times were considered; smeared and weak bands were excluded from the analysis. Dice’s coefficient of similarity (D_ij_) [39] was determined between each pair of strains. The estimates of similarity between strains were used for cluster analysis by the Unweighted Pair Group Method of Arithmetic Average (UPGMA [39]) using the NTSYS package version 2.02 for Windows [40]. The percentage of polymorphisms (Pp) was given as the number of polymorphic loci/number of total loci, regardless of allele frequencies.

### Culturing *B. subtilis* and FORL

*B. subtilis* strains were cultured in nutrient broth at 30°C with 180 rpm shaking in the dark for 48 h. The cell-free culture supernatant was collected by centrifugation at 6,000 × *g* for 15 min, and sequentially filtered through 0.45 µm and 0.22 µm organic filter membranes Shenzhen Landun Environmental Technology Co. Guangdong, China, respectively. The filtrate was used for the antimicrobial activity test. FORL was cultured as described previously [20].

### Inhibitory Activity of the Cell-free Supernatant of *B.* *subtilis* against FORL

To measure the inhibitory activity against mycelial growth, the cell-free supernatant prepared was added to agar plates (1.5% w/v agar) containing potato dextrose agar (PDA, Merck) to give a final concentration of 1.5, 3, 5, 10, 15, 20, 25, 30% (v/v). Nutrient broth (NB, Merck) was used as the control. Then, a 5-mm mycelial plug was removed from the margin of the FORL colony and placed in the center of the PDA plate. Plates were incubated at 28°C for 4 days and examined for fungal growth. The inhibition activity was expressed in terms of percentage of mycelial growth inhibition and was calculated according to the following formula: Inhibition (%) = [(Growth in control – Growth in treatment)/Growth in control] × 100.

### Measuring Effect of Temperature and Enzymes on Inhibiton

An inhibitory bioassay was carried out as described previously [41]. The effects of temperature on the cell-free supernatant prepared were tested by incubating cell-free supernatants at 60, 90 and 100°C for 1 h and at 121°C for 30 min. To determine the sensitivity of the active substance to proteolytic and other enzymes, 1 mL of the cell-free filtrate was added to 1 mg·mL^−1^ trypsin, 1 mg·mL^−1^ pepsin, 1 mg·mL^−1^ papain and 1 mg·mL^−1^ Proteinase K, respectively. Then, samples were incubated at 37°C for 30 min and then heated at 95°C for 5 min. After treatments, a 50-µL aliquot of cell-free culture supernatant was spotted onto an agar plate (1.5% (w/v) agar) seeded with the FORL spores (approx. 10^8^ CFU/mL) [42]. The cell-free supernatant without treatment and water were used as the control. Plates were incubated at 28°C in the dark for 48–72 h. The residual activity against FORL after treatments was tested by the well diffusion assay [43].

### Determining Antifungal Activities of Volatiles from *B.* *subtilis* against FORL

A bioassay was performed in sealed dishes using a previously described method [43] with some modifications. Briefly, 300 µL of *B. subtilis* (QST 713, EU07 and FZB24) cultures were spread onto a sterile plate containing TYB medium (g L^−1^) (tryptone 10, yeast extract 5, beef extract 3, glucose 20, KH_2_PO_4_ 0.5, Mg_2_SO_4_ 0.3, MnSO_4_ 0.07, Fe_2_SO_4_, citric acid 0.3, agar 1.5, pH 7.2). A 5-mm FORL mycelial plug taken from the margin of the colony was then placed in the centre of another new plate containing PDA. The fungal dish was immediately inverted over the bacterial dish and the dishes were rapidly sealed with Parafilm. The dishes were incubated at 28°C in the dark until the FORL mycelium of the controls extended to 3/4 of the plate. Volatiles from TYB medium instead of bacterial volatiles served as controls. The diameter (mm) of the fungal colony was measured.

### GC-MS Analysis of the Volatile Organic Compounds from *B. subtilis* Strains

Headspace solid phase microextraction (SPME) combined with gas chromatography-mass spectrometry (GC-MS) was used to analyse the volatile organic compounds (VOCs) from *B. subtilis* strains as described previously [44]. Each sample independently was analysed two times. Briefly, *B. subtilis* strains were grown in 20-ml vials sealed with parafilm on TYB liquid medium for 24 h at 30°C before collection of VOCs. The sample vials were placed on a heat stirrer at 50°C with an SPME fiber (50/30 µm DVB/CAR on PDMS, Agilent) inserted into the headspace of the sample vials for 40 min. Then SPME fibers were desorbed at 210°C for 1 min in the injection port of an Agilent 6890N-5975 GC/MS (Agilent Technologies, US) with a DB-5 column (30 m×0.25 mm×0.25 µm). GC/MS runs were 34.67 min and the fibers were conditioned at 210°C for 10 min before re-use. The injection port was in a splitless mode with a constant He flow of 1.0 ml min^−1^. The initial oven temperature was 50°C, held for 3 min, ramped at 6°C min^−1^ to 180°C and ramped at 10°C min^−1^ to 250°C and held for 3 min. The temperature of the transfer line and ion trap were 200°C and 230°C, respectively, with a continuous scan from 30 to 450 m/z.

### Voges-Proskauer Assay for Acetoin Production

*B. subtilis* (QST 713, EU07 and FZB24) was cultured in nutrient broth at 30°C for 48 h. The supernatants were collected by centrifugation at 12,000 rpm for 15 min and used for the Voges-Proskauer test [45] in which 1.0 mL of supernatant was transfered to a glass test tube, and then 0.6 mL of 5% α-naphtho, (Sigma, Aldrich) was added, followed by 0.2 mL of 40% KOH. The reactions were mixed after each addition and the color was allowed to develop at room temperature for 30 min.

### Two-dimensional Gel Electrophoresis Analysis of *B.* *subtilis* EU07 and FZB24

*B. subtilis* EU07 and FZB24 were grown at 30°C under vigorous agitation in NB medium. Cells were harvested in the late mid-exponential phase by centrifugation at 6,000×*g* for 15 min, then washed twice with TE (0.1M Tris-HCl, pH 7.5, 1 mM EDTA). The cells were suspended in 4 mL of lysis buffer (40 mM Tris-base, 7 M urea, 2 M thiourea, 2% w/v CHAPS, 1 mM EDTA, 1 mM PMSF, 50 mM DTT) and disrupted by ultrasonication (15 Hz, 5×30 s bursts with 1-min breaks) on ice. The supernatant was collected by centrifugation at 16,000×*g* for 15 min at 4°C and kept as a crude protein sample. To remove non-protein material from the extract and to determine the final protein concentration, a 2D Clean-up Kit (GE Healthcare, UK) and a 2D Quant Kit (GE Healthcare) were used sequentially according to the manufacturer’s instructions.

Proteins were separated by 2-D electrophoresis as described previously [46]. Briefly, 600 µg of proteins was dissolved in rehydration buffer (7 M urea, 2 M thiourea, 4% w/v CHAPS, 40 mM DTT, 0.5% (v/v) pH 4–7 IPG buffer and 0.0025% bromophenol blue) and rehydrated into immobilized pH 4–7 IPG strips (linear gradient, 24 cm; Amersham Biosciences, Uppsala, Sweden). Rehydration and isoelectric focusing (IEF) were carried out on an Ettan IPGphor II apparatus (Amersham Biosciences) under the step-n-hold mode as follows: 30 V for 12 h for reswelling, 500 V for 1 h, 1000 V for 1 h, and then voltage was increased to 8,000 V and kept constant at 8,000 V, resulting in a total of 80,000 Vh. Prior to SDS-PAGE, the strips were first equilibrated in 50 mM Tris–HCl pH 8.8, 6 M urea, 30% glycerol, 2% (w/v) SDS, 0.002% bromophenol blue containing 1% (w/v) DTT and subsequently with 4% (w/v) iodoacetamide, 15 min for each equilibration step, and then loaded onto 12.5% polyacrylamide gels using an Ettan™ DALTsix system (Amersham, Biosciences). Electrophoresis was performed at 1000 V 20 mA for 45 min and at 1000 V 40 mA for 2 h 40 min.

Proteins were visualised by Coomassie brilliant blue R-250 staining immediately after electrophoresis. The gray images were scanned using an ImageMaster TotalLab proteomic imaging system (Amersham Biosciences), and then spot detection, gel-to-gel matching, and spot quantification were carried out using 2-D electrophoresis gel analysis program ImageMaster 2D Platinum software 5.0 (Amersham Biosciences). The quantity of each spot was normalized with respect to the total spot volume detected in the gel. To examine the reliability of the data, only spots showing greater than 2.0-fold changes in expression were selected.

### Protein Identification by MALDI-TOF/TOF and Bioinformatics

Picking selected protein spots from the gels, in-gel digestion, extraction of tryptic peptides and spotting samples on Ettan MALDI target slides ready for MS analysis were carried out automatically with an Ettan Spot Handling Workstation (Amersham Biosciences). In the automated procedure, gel plugs were obtained by a 1.4-mm picking head, and washed twice in 50% methanol/50 mmol/lNH_4_HCO_3_ and once in 75% acetonitrile (ACN) before drying. For digestion, 10 µL of trypsin solution (0.02 µg/ml; sequencing grade, Promega) was added before incubation at 37°C for 2 h. Extraction was performed by adding 50% ACN and 0.1% trifluoroacetic acid (TFA) in two steps. The pooled extract was dried and dissolved in 3 µl matrix (5 mg/ml recrystallized α-cyano-4-hydroxycinnamic acid). Finally, 0.3 µl of dissolved sample was spotted on the target for MALDI-TOF-MS analysis. Identification of proteins and bioinformatic analyses of protein sequences were performed as described previously [47]. For MS analysis, PMF was obtained using Utraflex III MALDI-TOF/TOF (Bruker Daltonics Inc.) in the reflectron operation mode. All mass spectra were calibrated internally using trypsin (Promega Biosciences, CA) autolysis products at 842.509 Da and 2211.104 Da. Mass spectra were obtained over the m/z range of 700–3000 Da. Trypsin and keratin contamination peaks were excluded from the peak lists used in the database search. Each spectrum was produced by accumulating data from 200 consecutive laser shots. The results from the PMF were submitted to the MASCOT research engine (http://www.matrixscience.com), which was set to query the NCBI database (http://blast.ncbi.nlm.nih.gov/Blast.cgi) to obtain the corresponding protein identity.

### Liquid Chromatography Electrospray Ionisation Tandem Mass Spectrometry (LC-ESI-MS/MS) Analysis of Secretory Proteins from Strains EU07 and FZB24

Secreted proteins from the supernatants were extracted using the trichloroacetic acid (TCA) precipitation method previously described [44], and then separated by SDS-PAGE and visualized by Coomassie blue staining [46].

For protein identification, the bands of interest were excised from the gel and subjected to in-gel digestion and peptide extraction as described previously [47] with some modifications. Briefly, the gel bands were cut into small 1-mm^3^ cubes that were washed once with 500 µL of H_2_O, followed by three washes in 500 µL of 25 mM ammonium bicarbonate in 50% ACN for 60 min each on a mixer. The gel pieces were dehydrated following the addition of 500 µL ACN. Disulfide bonds were cleaved by incubating the samples for 60 min at 56°C with 200 µL of 10 mM DTT in 25 mM ammonium bicarbonate buffer. Alkylation of cysteines was performed by incubating the samples for 45 min at room temperature in darkness with 200 µL of 55 mM iodoacetamide in 25 mM ammonium bicarbonate buffer. The gel pieces were again dehydrated and the supernatant was discarded after each step. Gel pieces were covered with trypsin solution (10 ng/µL in 25 mM ammonium bicarbonate buffer). After 30 min incubation on ice, the remaining trypsin solution was removed, and 25 µL of 25 mM ammonium bicarbonate was added. Proteolysis was performed overnight at 37°C and stopped by adding 5% formic acid (FA) to the samples. The peptides in the gel were extracted once with 200 µL 0.1% FA in 50% ACN and twice by 200 µL 0.1% FA in 100% ACN. All supernatants were pooled and vacuum-dried.

### LC-ESI-MS/MS Analysis by LTQ-Orbitrap CID

The following step was modified from a previous study [47]. Each sample was resuspended in 40 µL buffer A (5% ACN, 0.1% FA) and centrifuged at 20,000×*g* for 10 min. All supernatants were loaded onto a Shimadzu LC-20AD nano HPLC by an autosampler onto a C18 trap column and the peptides were eluted onto a resolving 125 mm analytical C18 column (inner diameter 75 µm) packed in-house. The samples were loaded at 40 µL/min for 4 min, then a 40-min gradient was run at 400 nL/min starting from 5 to 35% B (95% ACN, 0.1% FA), followed by a 5-min linear gradient to 80%, maintained at 80% B for 4 min, and finally returned to 5% in 1 min.

The peptides were subjected to nanoelectrospray ionization followed by tandem mass spectrometry (MS/MS) in an Linear Trap Quadropole (LTQ) Orbitrap Velos (ThermoFisher Scientific, San Jose, CA) coupled online to the high performance liquid chromatography (HPLC). Intact peptides were detected in the Orbitrap at a resolution of 60000. Peptides were selected for MS/MS using the collision induced dissociation (CID) operating mode with a normalized collision energy setting of 35%; ion fragments were detected in the LTQ. A data-dependent procedure that alternated between one MS scan followed by 10 MS/MS scans was applied for the 10 most abundant precursor ions above a threshold ion count of 500 in the MS survey scan with the following Dynamic Exclusion settings: repeat counts, 2; repeat duration, 30 s; and exclusion duration, 120 s. The electrospray voltage applied was 1.5 kV. Automatic gain control (AGC) is used to prevent overfilling of the ion trap; 1×10^4^ ions were accumulated in the ion trap for generation of CID spectra. For MS scans, the m/z scan range was 300 to 1500 Da.

### Data Analysis and Interpretation

The instrument data file (.raw) was transformed to a mgf file by Proteome Discoverer (ver. 1.2.0.208; ThermoFisher Scientific, San Jose, CA). Peptide and protein identifications were performed using the Mascot search engine (ver. 2.3.0; Matrix Science, London, UK). Database searching was restricted to tryptic peptides of *B. subtilis* (downloaded from NCBI). Carbamidomethyl (C) was selected as fixed, deamidated (NQ), oxidation (M) as variable modifications, one missed cleavage allowed and precursor error tolerance at 10 mg L^−1^, fragment deviation at 0.6 Da. The complete list of identified peptides was then housed in an Excel (Microsoft, Redmond, WA) database for grouping of results into proteins [48]. The experiments were conducted as three independent replicates. Similar results were obtained in all replicates.

## Supporting Information

Figure S1 **Typical 2-DE protein profiles of B. subtilis EU07 and FZB24.** (A) EU07; (B) FZB24. The identified differentially expressed proteins are indicated by spot IDs on the gels (referenced to Table1). Horizontal axes are designated the pI and vertical axes the molecular mass.
(TIF)

Table S1 **Oligonucleotide primers used to detection of antibiotic genes.**
(DOCX)

Table S2 **List of proteins with altered expression identified by 2-DE/MALDI-TOF MS.**
(DOC)
