# Supplementary figures and images for: A Proteomic Approach Provides New Insights into the Control of Soil-Borne Plant Pathogens by Bacillus Species
Source: PLoS One. 2013 Jan 3;8(1):e53182. doi: 10.1371/journal.pone.0053182 (PMC3536778; doi:10.1371/journal.pone.0053182)

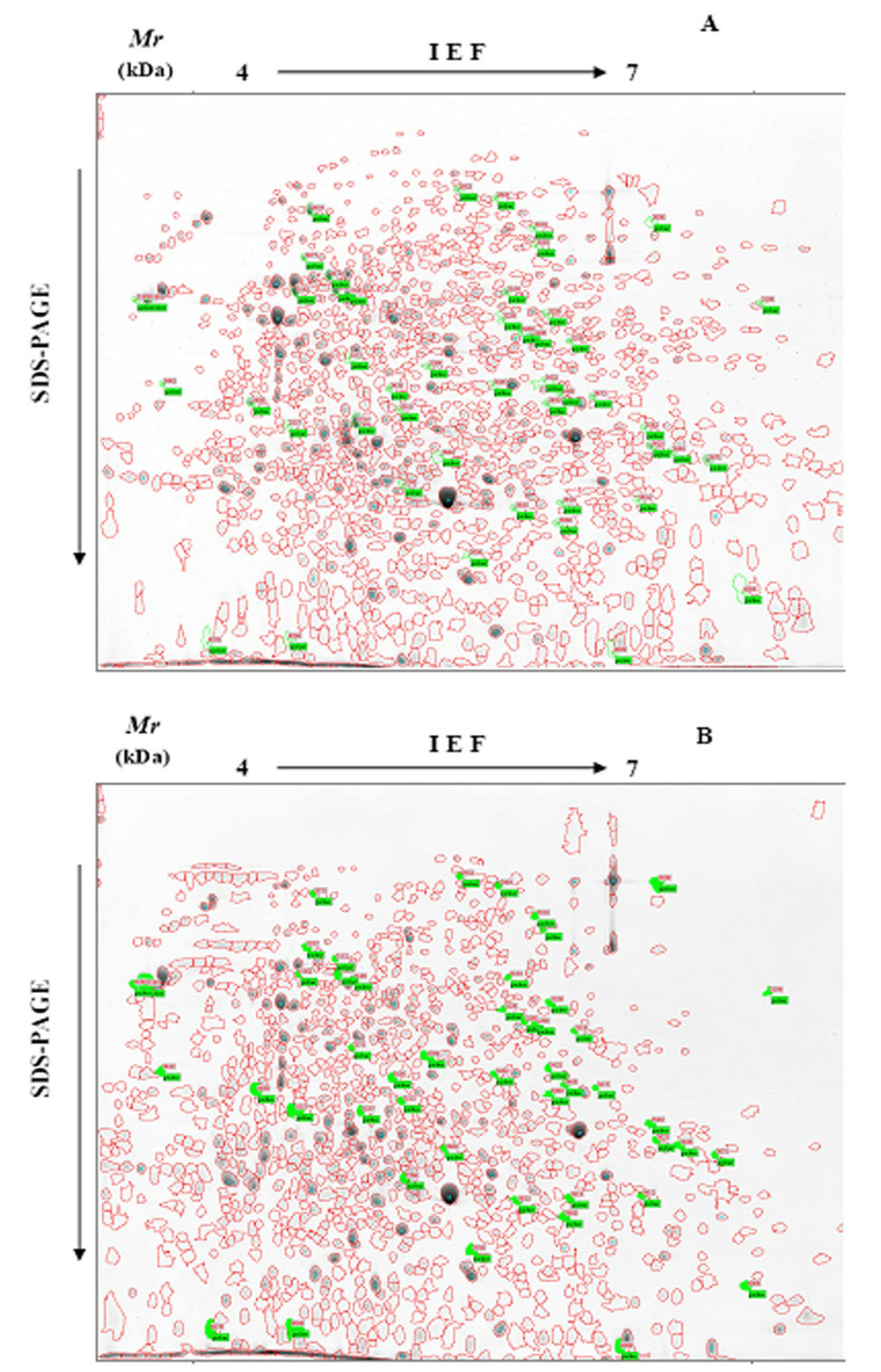

Supplement: Figure S1 — Typical 2-DE protein profiles of B. subtilis EU07 and FZB24. (A) EU07; (B) FZB24. The identified differentially expressed proteins are indicated by spot IDs on the gels (referenced to Table1). Horizontal axes are designated the pI and vertical axes the molecular mass. (TIF) [file pone.0053182.s001.tif]
