# Supplementary material for: A Proteomic Approach Provides New Insights into the Control of Soil-Borne Plant Pathogens by Bacillus Species
Source: PLoS One. 2013 Jan 3;8(1):e53182. doi: 10.1371/journal.pone.0053182 (PMC3536778; doi:10.1371/journal.pone.0053182)
Supplement: Table S1 — Oligonucleotide primers used to detection of antibiotic genes. (DOCX) [file pone.0053182.s002.docx]

| **Table S1. Oligonucleotide primers used to detection of antibiotic genes** | | |
| --- | --- | --- |
| **Primer** | | **Sequence** |
| Fengycin D | F | 5′-CCTGCAGAAGGAGGAGAAGTGAAG-3′ |
|  | R | 5′-TGCTCATCGTCTTCCGTTTC-3′ |
| Bacillomycin A | F | 5′-TGAAACAAAGGCATATGCTC-3′ |
|  | R | 5′-AAAAATGCATCTGCCGTTCC-3′ |
| Itu C | F | 5′-TTCACTTTTGATCTGGCGAT-3′ |
|  | R | 5′-CGTCCGGTACATTTTCAC-3′ |
