# Supplementary material for: A Proteomic Approach Provides New Insights into the Control of Soil-Borne Plant Pathogens by Bacillus Species
Source: PLoS One. 2013 Jan 3;8(1):e53182. doi: 10.1371/journal.pone.0053182 (PMC3536778; doi:10.1371/journal.pone.0053182)
Supplement: Table S2 — List of proteins with altered expression identified by 2-DE/MALDI-TOF MS. (DOC) [file pone.0053182.s003.doc]

| **Table S2.** List of proteins with altered expression identified by 2-DE/MALDI-TOF MS | | | | | | | | | | |
| --- | --- | --- | --- | --- | --- | --- | --- | --- | --- | --- |
| Spot ID^a^ | Up (↑) or down  (↓) regulated | Gene name | NCBI accession  no. | Protein Description | Function ^b^ | MS^c^ | M^d^ | SC^e^  (%) | p*I* | MW  (kDa) |
| 2952 | ↑ | Tkt | gi|16078852 | Transketolase | Carbohydrate metabolism | 127 | 8 | 17 | 4.99 | 72356 |
| 2964 | ↑ | yobO | gi|154686192 | YobO | unknown; similar to phage-related pre-neck appendage protein | 326 | 13 | 25 | 5.23 | 87081 |
| 2975 | ↑ | groEL | gi|16077670 | Chaperonin GroEL, Heat shock protein 60 family | Protein folding; Prevents misfolding and promotes the refolding and proper assembly of unfolded polypeptides generated under stress conditions | 444 | 22 |  | 4.53 | 57389 |
| 3000 | ↓ | clpC | gi|16077154 | Class III stress response-related ATPase , AAA+ superfamily | Protein folding;negative regulator of late competence genes; positive regulator of autolysin (LytC and LytD) synthesis; Competence gene repressor; required for cell growth at high temperature. Negative regulator of comK expression. May interact with mecA to negatively regulate comK. | 468 | 20 |  | 5.82 | 90120 |
| 3010 | ↑ | sdhA | gi|221310785 | Succinate dehydrogenase flavoprotein subunit | Carbohydrate metabolic, TCA cycle | 258 | 24 | 31 | 5.77 | 65539 |
| 3050 | ↑ |  | gi|221314158 | Alpha-ketoglutarate decarboxylase | Carbohydrate metabolic | 167 | 10 | 13 | 5.78 | 106030 |
| 3077 | ↑ | atpD | gi|16080734 | F0F1 ATP synthase subunit beta | Energy production and conversion | 344 | 14 | 32 | 4.80 | 51388 |
| 3112 | ↑ | rpsA | gi|16079345 | 30S ribosomal protein S1 | Translation, Metabolism of nucleotides and nucleic acids | 253 | 11 | 29 | 4.76 | 42376 |
| 3149 | ↑ | pepT | gi|16080943 | peptidase T | Cleaves the N-terminal amino acid of tripeptides; proteolysis; peptide metabolic process | 96 | 2 | 3 | 4.73 | 45538 |
| 3162 | ↑ | tufa | gi|16077181 | elongation factor Tu | Translation; This protein promotes the GTP-dependent binding of aminoacyl-tRNA to the A-site of ribosomes during protein biosynthesis | 350 | 12 | 27 | 4.92 | 43680 |
| 3164 | ↑ | rocD | gi|154688124 | ornithine--oxo-acid transaminase | Amino acid metabolism; Catalyzes the interconversion of ornithine to glutamate semialdehyde. Controls arginine catabolism | 590 | 19 | 48 | 5.27 | 44045 |
| 3168 | ↑ | Icd | gi|16079965 | isocitrate dehydrogenase | Carbohydrate metabolic; The enzyme can be phosphorylated in vitro by the E.coli isocitrate dehydrogenase kinase/phosphatase, but B.subtilis lacks such an enzyme. | 123 | 9 | 19 | 5.03 | 46503 |
| 3180 | ↓ |  | gi|1139531 | 90k-protease (bacillopeptidase F) | Protein folding and degradation | 57 | 3 | 1 | 5.14 | 154481 |
| 3189 | ↓ |  | gi|1139531 | 90k-protease (bacillopeptidase F) | Protein folding and degradation | 54 | 3 | 1 | 5.14 | 154481 |
| 3199 | ↑ |  | gi|193506559 | Chain A, Crystal Structure Of Gamma-Glutamyl Transferase | Amino acid metabolism | 90 | 4 | 12 | 5.38 | 41376 |
| 3229 | ↑ | gapA | gi|16080447 | glyceraldehyde-3-phosphate dehydrogenase | Carbohydrate metabolic; More active in catabolism. | 121 | 5 | 19 | 5.20 | 35924 |
| 3238 | ↑ | gapA | gi|16080447 | glyceraldehyde-3-phosphate dehydrogenase | Carbohydrate metabolic | 276 | 7 | 20 | 5.20 | 35924 |
| 3280 | ↑ | atpA | gi|16080736 | F0F1 ATP synthase subunit alpha | Energy production and conversion; Produces ATP from ADP in the presence of a proton gradient across the membrane. The alpha chain is a regulatory subunit | 124 | 8 | 12 | 5.22 | 54679 |
| 3296 | ↑ | atpA | gi|16080736 | F0F1 ATP synthase subunit alpha | Energy production and conversion | 292 | 9 | 16 | 5.22 | 54679 |
| 3318 | ↑ | yqfO | gi|154686778 | YqfO | function unknown(Belongs to the UPF0135 (NIF3) family.) | 228 | 14 | 39 | 5.61 | 41221 |
| 3371 | ↑ | Mdh | gi|16079964 | malate dehydrogenase | Carbohydrate metabolic | 173 | 9 | 34 | 4.92 | 33623 |
| 3394 | ↑ | Amps | [gi|16078509](http://202.116.111.25/mascot/cgi/protein_view.pl?file=../data/20100324/F005887.dat&hit=1) | aminopeptidase | Amino acid metabolism; Belongs to the peptidase M29 family. | 82 | 7 |  |  | 45770 |
| 3442 | ↓ | clpC | gi|16077154 | class III stress response-related ATPase | Protein folding | 33 | 6 |  |  | 90120 |
| 3446 | ↑ | cysK | gi|16077141 | cysteine synthetase A | Sulphur amino acid biosynthesis; Catalyzes the conversion of O-acetylserine to cysteine. Also acts as a sensor of cysteine availability in the signal transduction pathway modulating cymR activity. When cysteine is present, the pool of O-acetylserine (OAS) is low, which leads to the formation of a cymR-cysK complex and transcriptional repression of the cymR regulon occurs. In the absence of cysteine, the OAS pool is high and the cymR-cysK complex is mostly dissociated, leading to a faster dissociation of cymR from its DNA targets and the lifting of cymR-dependent repression | 87 | 6 | 22 | 5.64 | 32799 |
| 3456 | ↑ | gtaB | gi|16080620 | UTP-glucose-1-phosphate uridylyltransferase | Amino sugar and nucleotide sugar metabolism, glucosylation of teichoic acid; Catalyzes the formation of UDP-glucose from glucose-1-phosphate and UTP. This is an intermediate step in the biosynthesis of diglucosyl-diacylglycerol (Glc2-DAG), i.e. the predominant glycolipid found in *B .subtilis* membrane, which is also used as a membrane anchor for lipoteichoic acid (LTA). Has a role in the biosynthesis of all phosphate-containing envelope polymers, since UDP-glucose serves as a glucosyl donor not only for the biosynthesis of LTA but also for wall teichoic acids (WTAs). Is required for biofilm formation. This is likely due to another role of UDP-glucose, which might also act as a metabolic signal regulating biofilm formation or may be involved in some unknown biosynthetic pathway essential for biofilm formation, e.g. the synthesis of an exopolysaccharide | 101 | 6 | 24 | 5.10 | 33106 |
| 3468 | ↑ | metE | gi|16078383 | 5-methyltetrahydropteroyltriglutamate--homocysteine methyltransferase | Sulphur amino acid biosynthesis; Catalyzes the transfer of a methyl group from 5-methyltetrahydrofolate to homocysteine resulting in methionine formation | 26 | 1 | 1 | 5.20 | 87003 |
| 3475 | ↑ | yqfS | gi|16079568 | endonuclease IV | DNA repair; Endonuclease IV plays a role in DNA repair. It cleaves phosphodiester bonds at apurinic or apyrimidinic sites (AP sites) to produce new 5'-ends that are base-free deoxyribose 5-phosphate residues. It preferentially attacks modified AP sites created by bleomycin and neocarzinostatin | 181 | 6 | 16 | 5.47 | 33219 |
| 3488 | ↓ | Vpr | gi|157850259 | extracellular protease vpr | Metabolism of amino acids and related molecules; not required for growth or sporulation | 101 | 1 | 1 | 5.56 | 66104 |
| 3490 | ↑ | menB | gi|143186 | dihydroxynapthoic acid (DHNA) synthetase | similar to naphthoate synthase ；Converts o-succinylbenzoyl-CoA (OSB-CoA) to 1,4-dihydroxy-2-naphthoic acid (DHNA).; Cofactor biosynthesis; menaquinone biosynthesis ; ubiquinone and other terpenoid-quinone biosynthesis | 277 | 5 | 22 | 5.44 | 28815 |
| 3513 | ↓ | yobO | gi|154686192 | YobO | unknown; similar to phage-related pre-neck appendage protein | 169 |  | 23 | 5.23 | 87081 |
| 3537 | ↑ | Hag | gi|124502416 | flagellin | Motility and chemotaxis; Flagellin is the subunit protein which polymerizes to form the filaments of bacterial flagella | 219 | 7 | 21 | 4.93 | 33666 |
| 3562 | ↑ |  | gi|76667926 | two component response regulator | signal transduction | 90 | 10 | 47 | 5.66 | 25859 |
| 3621 | ↑ |  | gi|50812247 | alpha-ketoglutarate decarboxylase | Carbohydrate metabolic | 105 | 6 | 8 | 5.91 | 105902 |
| 3646 | ↑ |  | gi|76801599 | monooxygenase(homolog to alkanesulfonate monooxygenase) | Redox regulation | 67 | 1 | 3 | 4.28 | 36669 |
| 3662 | ↑ | fusA | gi|221307924 | elongation factor G | Translation | 124 | 7 | 8 | 4.80 | 76740 |
| 3671 | ↑ | glyA | gi|16080743 | serine hydroxymethyltransferase | Amino acid metabolism | 119 | 5 | 13 | 5.56 | 45575 |
| 3766 | ↓ | fbaA | gi|16080765 | fructose-bisphosphate aldolase | Carbohydrate metabolic | 115 | 8 | 38 | 5.19 | 30552 |
| 3812 | ↑ | yhfR | gi|154685486 | YhfR | Carbohydrate metabolic, similar to 2,3-diphosphoglycerate-dependent phosphoglycerate mutase | 275 | 13 | 53 | 5.64 | 21506 |
| 3819 | ↑ | yuaE | gi|16080155 | hypothetical protein | function unknown and unique | 60 | 3 |  | 5.49 | 19099 |
| 3835 | ↑ | yuaE | gi|154687215 | YuaE | function unknown and unique | 300 | 14 | 72 | 5.46 | 19112 |
| 3866 | ↑ |  | gi|154685032 | hypothetical protein | function unknown | 200 | 12 | 52 | 5.39 | 29129 |
| 4089 | ↓ | fusA | gi|221307924 | elongation factor G | Translation | 107 | 7 | 14 | 4.80 | 76740 |
| a) Numbering corresponds to the 2-DE gel in Fig. 7  b) Functional annotations are taken from the *Bacillus subtilis* Genome Database (http://genolist.pasteur.fr/SubtiList/index.html;  http://bacillus.genome.ad.jp/bsorf.html).  c) MS, Mascot score.  d) M, number of peptides matched  e) SC, sequence coverage by peptide mass fingerprinting using MALDI-TOF | | | | | | | | | | |
